# Evaluation of a gp63–PCR Based Assay as a Molecular Diagnosis Tool in Canine Leishmaniasis in Tunisia

**DOI:** 10.1371/journal.pone.0105419

**Published:** 2014-08-25

**Authors:** Souheila Guerbouj, Fattouma Djilani, Jihene Bettaieb, Bronwen Lambson, Mohamed Fethi Diouani, Afif Ben Salah, Riadh Ben Ismail, Ikram Guizani

**Affiliations:** 1 Laboratory of Molecular Epidemiology and Experimental Pathology, Pasteur Institute of Tunis, Université de Tunis el Manar, Tunis, Tunisia; 2 Laboratory of Epidemiology and Ecology of Parasitic Diseases, Pasteur Institute of Tunis, Tunis, Tunisia; 3 Laboratory of Medical Epidemiology, Pasteur Institute of Tunis, Tunis, Tunisia; 4 Molteno Institute for Parasitology, Department of Pathology, University of Cambridge, Cambridge, United Kingdom; Queensland Institute of Medical Research, Australia

## Abstract

A gp63PCR method was evaluated for the detection and characterization of *Leishmania (Leishmania)* (*L.*) parasites in canine lymph node aspirates. This tool was tested and compared to other PCRs based on the amplification of 18S ribosomal genes, a *L. infantum* specific repetitive sequence and kinetoplastic DNA minicircles, and to classical parasitological (smear examination and/or culture) or serological (IFAT) techniques on a sample of 40 dogs, originating from different *L. infantum* endemic regions in Tunisia. Sensitivity and specificity of all the PCR assays were evaluated on parasitologically confirmed dogs within this sample (N = 18) and control dogs (N = 45) originating from non–endemic countries in northern Europe and Australia. The gp63 PCR had 83.5% sensitivity and 100% specificity, a performance comparable to the kinetoplast PCR assay and better than the other assays. These assays had comparable results when the gels were southern transferred and hybridized with a radioactive probe. As different infection rates were found according to the technique, concordance of the results was estimated by (κ) test. Best concordance values were between the gp63PCR and parasitological methods (74.6%, 95% confidence intervals CI: 58.8–95.4%) or serology IFAT technique (47.4%, 95% CI: 23.5–71.3%). However, taken together Gp63 and Rib assays covered most of the samples found positive making of them a good alternative for determination of infection rates. Potential of the gp63PCR-RFLP assay for analysis of parasite genetic diversity within samples was also evaluated using 5 restriction enzymes. RFLP analysis confirmed assignment of the parasites infecting the dogs to *L. infantum* species and illustrated occurrence of multiple variants in the different endemic foci. Gp63 PCR assay thus constitutes a useful tool in molecular diagnosis of *L. infantum* infections in dogs in Tunisia.

## Introduction

Visceral leishmaniasis due to *Leishmania infantum* is endemic in Mediterranean basin countries, Middle East, Latin America and Asia. Canines are the major reservoir of the infection [1]. Infected dogs present either a range of clinical manifestations of a viscero-cutaneous form or an asymptomatic status. These latter are considered as carriers since they are for sand flies as infectious as the symptomatic ones [2], [3]. Canine leishmaniasis (CanL) is a major veterinary and public health problem not only in old endemic foci but also in non endemic areas where outbreaks are occasionally reported, such as in the United States and Canada [4] and in northern Europe [5]. For epidemiological purposes, there is a need for a precise estimation of the number of infected dogs to evaluate the real extent of infection and better elaborate control programs. Several diagnostic techniques are available for detection of canine infection or diagnosis of the disease. These can be achieved either by demonstrating the parasites microscopically in stained smears [6] or after *in vitro* cultivation [7]. Indirect methods use mainly serological means, like the enzyme-linked immunosorbant assay (ELISA) [8], [9], the indirect immunofluorescence assay (IFAT) [10], [11] and the direct agglutination test (DAT) [12], [13]. However, these diagnostic methods present limitations essentially due to their sensitivity and specificity: parasitological techniques are characteristically insensitive and serological tests are limited by their inability to distinguish between past and present infections and the possibility of cross-reactions with other infectious agents [8], [14], [15]. With the advent of DNA-based methods and the polymerase chain reaction particularly, more sensitive and rapid detection of parasites has become possible. Although several groups have tested PCR assays in different types of biological samples (fresh, frozen, formalin-fixed or paraffin-embedded biopsies) for the detection of *Leishmania* [9], [16], [17], [18], their values in diagnosis of canine leishmaniasis were partially evaluated.

Here we evaluate a gp63PCR-based technique [19] for molecular diagnosis of *Leishmania* infection in dogs collected from *L. infantum* stable transmission areas in Tunisia, comparing it to classical parasitological and serological techniques and to other molecular assays based on PCR amplification of 18S ribosomal genes, an *L. infantum* specific repetitive sequence and kinetoplast DNA minicircles.

## Materials and Methods

### Ethics statement

All canine sampling was conducted during routine veterinary care in primary practices. Sampling of Tunisian dogs was conducted during a previous survey study within leishmaniasis endemic regions in Tunisia [20], where (with the exception of 7 dogs that were received at the clinic of the veterinary school for diagnosis) stray dogs were collected during campaigns performed jointly by the Ministries of health and of the Interior, integrating analysis of *Leishmania* prevalence to anti-rabies control programs (stray dog culling). At the time of the study as the veterinarians of our institute took care of these dogs under humane conditions, we did not request ethical consent from the recently installed ethics committee at our institution, to take and use samples of stray dogs that were caught for elimination, or dog samples taken to confirm diagnosis of clinically patent dogs. We used in this last case, remains of the aspirate taken for culture to extract DNAs. Nevertheless, collection of dogs was performed in compliance with the directive 86/609/EEC of the European parliament and the council on the protection of animals used for scientific purposes, in agreement with the guidelines of International Guiding Principles for Biomedical Research Involving Animals.

The second group is composed of 45 control dogs living in regions free from leishmaniasis in northern Europe and Australia (Table S2). Extracted DNAs from samples of these dogs were kindly provided by Dr. David Sargan (University of Cambridge, UK). Sampling of these control dogs was in a range of clinical tests. In all cases the owners gave informed consent that any excess of samples taken during clinical testing could be used in research so long as that excess formed a minority of the sample. This was in accordance with UK Home Office Guidelines. Dogs were not anesthetized. The samples represent, in the case of the Cardigan Welsh Corgis and the Irish setter, DNA from excess blood (<1 ml) after a DNA based test for the rcd3 and rcd1 PRA mutations, respectively. The blood (2–5 ml) was collected as clinical samples into EDTA tubes without anesthesia. This was done by veterinary surgeons in primary practices. In the case of the Irish wolfhound, surplus blood (<1 ml) from clinical collection (usually 2 ml) was taken by a veterinary surgeon for blood ammonia and other tests as part of work up in surveillance for portosystemic shunt. Collections took place at a number of referral clinics. In the cases of the Cocker spaniel and Labrador retriever these were excess (<1 ml) from cases where EDTA blood, usually 2–5 ml, was collected for routine hematological work up in the clinics of the Queen's Veterinary Hospital, University of Cambridge.

### Dogs and samples

Two groups of dogs were studied. The first group corresponded to a total of 40 dogs, from endemic areas of leishmaniasis in Tunisia (Table S1). Lymph node aspirates (∼100 µl) were taken by veterinary surgeons in primary practices and stored at −80°C before DNA extraction. Dogs were not anesthetized and sampling did not make any suffering. This dog group has been previously characterized in our laboratory, using parasitological (Giemsa stained smear examination and in vitro culture), serological (IFAT) and molecular (PCR) tests (Table S1). Only seven dogs (J1– J7) from this group have acute leishmaniasis that presented with clinical and biological features of the disease to the clinic of Sidi Thabet Veterinary school (Tunisia). The remaining 33 dogs did not have patent leishmaniasis; some of them were however oligosymptomatic. The 18 dogs that were positive either by smear examination or culture inoculation (parasitologically confirmed dogs) constituted the positive control group.

The second group is composed of 45 control dogs living in regions free from leishmaniasis in northern Europe and Australia (Table S2). Extracted DNAs from samples of these dogs were kindly provided by Dr. David Sargan (University of Cambridge, UK). Details about the samples are provided in the ethics statement section.

### DNA extraction

Frozen lymph node aspirates were suspended in a lysis solution (50 mM NaCl, 10 mM EDTA, 50 mM Tris–HCl pH 7.4) and incubated overnight at 55°C with 100 µg/ml Proteinase K and 0.05% SDS. Total DNA was phenol/chloroform purified and ethanol precipitated as previously described [21].

### PCR amplification

Different PCR tests were applied to dog DNA samples. The first PCR targets the coding region of gp63 genes of *Leishmania*, using specific primers SG1 and SG2 as previously described [19]. The second PCR amplifies a central region of a ribosomal gene encoding for the 18S subunit (RIB PCR) present in all *Leishmania* parasites [22]. The third PCR used in this study targets a repetitive genomic sequence found in *L. infantum* species (INF PCR, Genebank Accession No. L42486.1) [23]. KIN PCR used primers KINF and KINR to amplify minicircles of the kinetoplastic DNA of *Leishmania* [24]. Table 1 summarizes the primers sequences used for the different PCRs and the amplified fragments sizes expected. Reaction and cycling conditions are also presented on Table 1.

**Table 1:** PCR primers sequences, reaction and cycling conditions used.

PCR code	Target	Forward primer Sequence (5′ – 3′)	Reverse primer sequence (5′ – 3′)	Amplified fragment size (bp)	Annealing temperature (°C)	Agarose gel percentage	Reaction conditions (25 µl final volume)	Cycling conditions
**Gp63**	Gp63 gene family	SG1: GTCTCCACCGAGGACCTCACCGA	SG2: TGATGTAGCCGCCCTCCTCGAAG	1300	65	1,2%	22,5 pmol each primer; 0,2 mM dNTPs; 1 mM MgCl_2_; 0,5 units Taq DNA polymerase (PerkinElmer, France); 50 ng template DNA; 5% DMSO	94°C: 5 min; 35 cycles of 94°C: 30 sec; annealing: 30 sec; 72°C: 1 min; 72°C: 10 min
**RIB**	18S ribosomal gene	RIBF: GGTTCCTTTCCTGATTTACG	RIBR: GGCCGGTAAAGGCCGAATAG	650	60	1,6%	22,5 pmol each primer; 0,2 mM dNTPs; 1 mM MgCl_2_; 0,5 units Taq DNA polymerase (PerkinElmer, France); 50 ng template DNA	94°C: 5 min; 35 cycles of 94°C: 30 sec; annealing: 30 sec; 72°C: 1 min; 72°C: 10 min
**INF**	Repetitive genomic sequence	INFF: ACGAGGTCAGCTCCACTCC	INFR: CTGCAACGCCTGTGTCTAC	100	59	2%	22,5 pmol each primer; 0,2 mM dNTPs; 1 mM MgCl_2_; 0,5 units Taq DNA polymerase (PerkinElmer, France); 50 ng template DNA	94°C: 5 min; 35 cycles of 94°C: 30 sec; annealing: 30 sec; 72°C: 1 min; 72°C: 10 min
**KIN**	Minicircles of the kinetoplastic DNA	KINF: GGGGTTGGTGTAAAATAGGGCCGG	KINR: CCAGTTTCCCGCCCCGGAG	800	67	1,5%	22,5 pmol each primer; 0,2 mM dNTPs; 1 mM MgCl_2_; 0,5 units Taq DNA polymerase (PerkinElmer, France); 50 ng template DNA	94°C: 5 min; 35 cycles of 94°C: 30 sec; annealing: 30 sec; 72°C: 1 min; 72°C: 10 min
**PO**	Acidic ribosomal phosphoprotein	POF: TCATTGTGGGAGCAGACA	POR: GGAGAAGGGGGAGATGTT	470	51	1,5%	20 pmol each primer; 0,2 mM dNTPs; 1,5 mM MgCl_2_; 1,25 units Taq DNA polymerase (PerkinElmer, France); 20 ng template DNA	94°C: 5 min; 35 cycles of 94°C: 30 sec; annealing: 30 sec; 72°C: 1 min; 72°C: 10 min

Another PCR used in this study amplifies a dog gene (acidic ribosomal phosphoprotein fragment, PO) in order to assess for possible sample degradation prior to analysis or inhibition of amplification. PO primers (Table 1), designed from human, rat and mouse PO gene sequences, cross-react with dog DNA and allow amplification of a 470 bp fragment [25]. Products from the different PCRs were analyzed by agarose gel electrophoresis.

In all PCR reactions, multiple negative controls (no DNA) were included in order to monitor for possible contamination. Furthermore, to avoid contamination of samples during carryover and processing, separated laboratory spaces were used for PCR reaction preparation and for analysis of amplified products (gel preparation and migration). Filter-filled tips were also used to set up the PCR reactions. All results were confirmed by hybridization to specific radio-labeled probes.

### Probes and hybridization

All PCR gels were transferred onto Hybond N+ membranes according to the Southern method [21] and hybridized to specific probes at 65°C. RIB, INF and KIN PCRs unique fragments (650 bp, 100 bp and 800 bp, respectively) amplified from a positive control corresponding to an *L. infantum* DNA (MHOM/TN/96/Drep15), were gel-extracted, purified using the Qiaquick gel extraction kit (Qiagen, Paris, France) and used as probes. Whereas, a 2 kb fragment corresponding to the coding region of the *L. infantum* gp63 gene was used for gp63PCRs, as previously described [19]. Probes were labeled with α^32^P dCTP using the random primer labeling kit (Amersham–HVD, Athens, Greece) and used to hybridize blots of all the gels. After high stringency washes, labeled hybrid DNA was visualized on X–ray sensitive auto-radiographic films.

### Gp63 PCR-RFLP and cluster analysis

Amplified gp63 fragments were digested with *BsiE*I, *Msc*I, *Hinc*II, *BsmB*I and *Sal*I restriction enzymes (Amersham–HVD, Athens, Greece) as previously described [19]. PCR-RFLP profiles were analyzed after overnight electrophoresis in 3% agarose gels and subsequently hybridized to the ^32^P-labelled gp63 probe. Restriction bands obtained with all the restriction enzymes were scored 1 or 0 for presence or absence of bands, respectively. Genetic distances according to the Nei-modified method were calculated from RFLP data. This data served to construct a dendrogram according to the Kitch method [26], using the PHYLIP package (version 3.69). The Kitch program constructs a tree by successive (agglomerative) clustering, using an average-linkage method of clustering, similar to that used in the UPGMA method. However, this method was chosen as it assumes a molecular clock, allowing the total length of branches from the root to any species to be the same. In addition, this program has options that allow after the tree is constructed to remove and re-add each group, and to try alternative topologies, thus improving the result [26].

### Concordance test

The concordance between results of the parasitological, serological or molecular tests was estimated by determining the kappa coefficient (95% CI) using the kappa (k) test of concordance [27]. The Kappa coefficient is interpreted in accordance with Landis and Koch [28] as almost perfect (1.00–0.81), substantial (0.80–0.61), moderate (0.60–0.41), fair (0.40–0.21) and slight (0.20–0.0). The statistical analysis was carried out using the SPSS software package (Version 13.0).

## Results

### Assessment of DNA quality by PO PCR

The PO primers expected to amplify a 470 bp fragment of a mitochondrial phosphoprotein gene present in mammals [25] were used to evaluate occurrence of inhibition during amplification in dog samples DNAs. All the 40 Tunisian dogs'DNAs generated the expected fragment size of 470 bp (Table 2 and Table S1). Among the control group (N = 45), 3 DNAs were negative (Table 2 and Table S2), indicating PCR inhibition or DNA degradation.

**Table 2:** Results of parasitology, serology and PCR investigations on Tunisian and control dogs.

		Parasitology	Serology	PO PCR^a^	RIB PCR^b^		INF PCR^b^		KIN PCR^b^		gp63 PCR^b^	
					EtBr	^32^P	EtBr	^32^P	EtBr	^32^P	EtBr	^32^P
**Tunisian dogs (N = 40)**	Positive results	18/40	26/40	40/40	26/40	35/40	24/40	39/40	20/40	33/40	17/40	17/40
	Sensitivity^c^ (%)				55.6 (10/18)	83.5 (15/18)	83.5 (15/18)	100 (18/18)	61.1 (11/18)	83.5 (15/18)	83.5 (15/18)	83.5 (15/18)
	Infection rate^d^ (%)	45.0 (18/40)	65.0 (26/40)			87.5 (35/40)		97.5 (39/40)		82.5 (33/40)		42.5 (17/40)
**Control dogs (N = 45)**	Positive results			42/45	0/45	3/45	0/45	5/45	0/45	0/45	0/45	1/45
	Specificity^e^ (%)				100	93.3 (42/45)	100	88.9 (40/45)	100	100	100	97.8 (44/45)

a PO PCR targets a mammalian mitochondrial phosphoprotein gene.

b RIB, INF, KIN and gp63 PCRs target a central region of 18S ribosomal gene, a repetitive genomic sequence, minicircles of the kinetoplastic DNA and gp63 family coding sequences, respectively in *Leishmania*.

c sensitivity of the different PCR assays corresponds to the proportion of positive dogs among the 18 parasitologically confirmed ones.

d infection rates are the proportion of positive dogs among the total dog number.

e specificity of the different PCR assays corresponds to the number of negative dogs among the control dog group.

Abbreviations: EtBr, Ethidium bromide staining and reading under UV light; ^32^P, autoradiographic reading after hybridization with a ^32^P labeled probe.

### *Leishmania* DNA PCR amplification from dog samples

The different PCR tests applied to the 85 dog DNA samples of the study showed that 26, 24, 20 and 17 dogs, all from the Tunisian group, were positive using RIB, INF, KIN and gp63 PCRs, respectively (Table 2) with fragments at the expected 650 bp, 100 bp, 800 bp and 1300 bp size, respectively. All control dogs, originating from non–endemic regions for leishmaniasis, did not present any amplification with the different PCR tests (Table 2).

In order to verify the specificity of the PCR products obtained and to assess the possibility of false negative results, all electrophoresis gels were Southern transferred onto a Nylon membrane and amplified products were hybridized to corresponding ^32^P labeled probes. Results obtained after Ethidium bromide (EtBr) staining and UV observation (before hybridization) and after probe hybridization were compared (Table 2 and Table S1). All bands observed on the gels were confirmed by the probe hybridizations. This step also increased the number of positive Tunisian dogs to 35, 39 and 33 with RIB, INF and KIN PCRs, respectively while the number of gp63 PCR positive dogs did not change after hybridization (Table 2).

### Sensitivity and Specificity of PCR tests

Sensitivity of the different PCR tests was measured as the proportion of positive dogs among the positive control group of 18 parasitologically confirmed dogs (Table 2). RIB, INF, KIN and gp63 PCRs had a sensitivity of 55.6% (10/18), 83.5% (15/18), 61.1% (11/18) and 83.5% (15/18), respectively. After ^32^P labeled probe hybridization, sensitivity changed to 83.5% (15/18), 100% and 83.5% (15/18) for RIB, INF, and KIN PCRs, respectively but it remained the same for gp63PCR (Table 2).

Specificity of the PCR tests was estimated as the proportion of the negative control dogs (originating from non–endemic regions for leishmaniasis) that were negative in the assays. 100% of specificity was achieved by all PCR tests after analysis of EtBr stained gels, while 93.3% (42/45), 88.9% (40/45) and 97.8% (44/45) were found for RIB, INF and gp63 PCRs, respectively, after autoradiography analysis (Table 2 and Table S2). This decrease was due to the presence of positive signals after hybridization in the case of several dogs (9/45) (Table S2). No change was observed for KIN PCR, after hybridization (Table 2).

### Comparative evaluation of parasitological, serological and molecular tools for diagnosis of canine leishmaniasis

Infection rate within our sample was estimated using the parasitological, serological and molecular techniques. Parasitological tests using direct examination of amastigotes within biopsies (stained smears) and *in vitro* isolation in culture media of promastigotes indicated a 45% (18/40) infection rate (Table 2). Using IFAT, the infection rate was 65% (26/40) (Table 2). With the PCR assays, considering the dogs were infected when positive signals were observed before or after hybridization, the infection rates reached 87.5% (35/40), 97.5% (39/40) and 82.5% (33/40) for RIB, INF and KIN PCRs, respectively while with the gp63PCR it was 42.5% (17/40) (Table 2). Given the differences in measures of infection rates, concordance of the results was investigated computing the proportion of identical results found by different tools and comparing them in a pair wise way (Table 3). The best concordance kappa (κ) values were found between the gp63PCR test and parasitological (74.6%, 95% confidence interval (CI): 0.588, 0.954) or serological IFAT (47.4%, 95%CI: 0.235, 0.713) methods (Table 3), with a substantial and moderate agreement between these tools, respectively. Concordance between the RIB, INF and KIN PCRs and parasitological or serological methods showed negative or close to zero (−0.304 to 0.041) kappa values, indicating a disagreement (Table 3). In addition, when the PCR tools were pair-wise compared, fair concordance kappa values were found between KIN and RIB (22%, 95%CI: −0.158, 0.598) and between KIN and INF (21.6%, 95%CI: −0.143, 0.575) PCRs (Table 3).

**Table 3:** Pair wise concordance values calculated using the kappa coefficient for parasitology, serology and PCR investigations.

		Parasitology		Serology (IFAT)		PCRs					
						RIB		INF		KIN	
		Kappa	95% CI*	Kappa	95% CI*	Kappa	95% CI*	Kappa	95% CI*	Kappa	95% CI*
**Serology (IFAT)**		0.515	0.274, 0.756								
**PCRs**	**RIB**	−0.070	−0.264, 0.124	−0.226	−0.387, −0.065						
	**INF**	0.041	−0.039, 0.121	−0.049	−0.141, 0.043	−0.043	−0.116, 0.029				
	**KIN**	0.014	−0.205, 0.233	−0.304	−0.473, −0.135	0.220	−0.158, 0.598	0.216	−0.143, 0.575		
	**gp63**	0.746	0.588, 0.954	0.474	0.235, 0.713	0.011	−0.169, 0.191	0.037	−0.035, 0.109	0.089	−0.117, 0.295

*CI, Confidence interval.

RIB, INF, KIN and gp63 PCRs target a central region of 18S ribosomal gene, a repetitive genomic sequence, minicercles of the kinetoplastic DNA and gp63 family coding sequences, respectively in *Leishmania*.

### Species identification and analysis of intra-specific parasite polymorphism by gp63 PCR-RFLP

**Figure 1:**
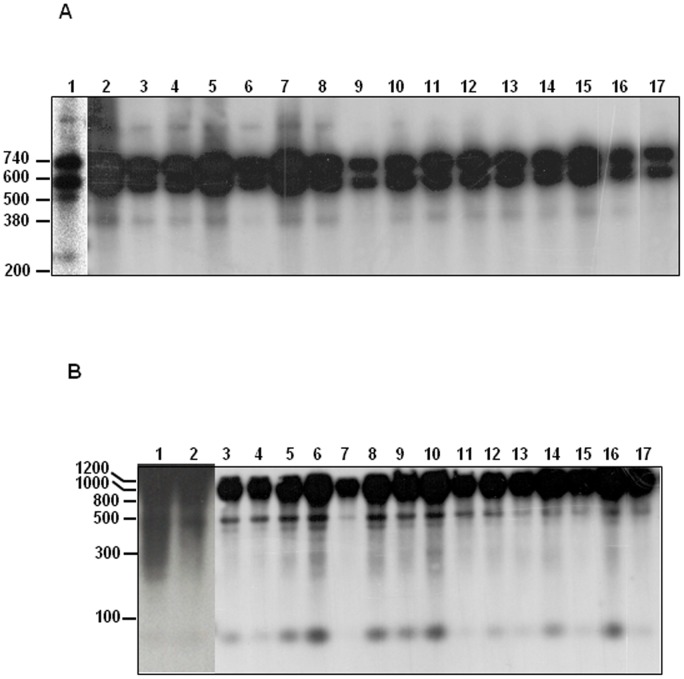
Gp63PCR-RFLP patterns of *Leishmania* parasites obtained from dog biopsies. A: digestion with *Msc*I restriction enzyme; B: digestion with *Sal*I restriction enzyme. 1: *L. donovani*, 2: *L. infantum*, 3: LN112, 4: LN129, 5: LN26, 6: LN11, 7: LN80, 8: LN2, 9: LN39, 10: LN77, 11: LN102, 12: LN110, 13: J1, 14: J3, 15: J5, 16: J6, 17: J7. All sizes are indicated in bp.

**Figure 2:**
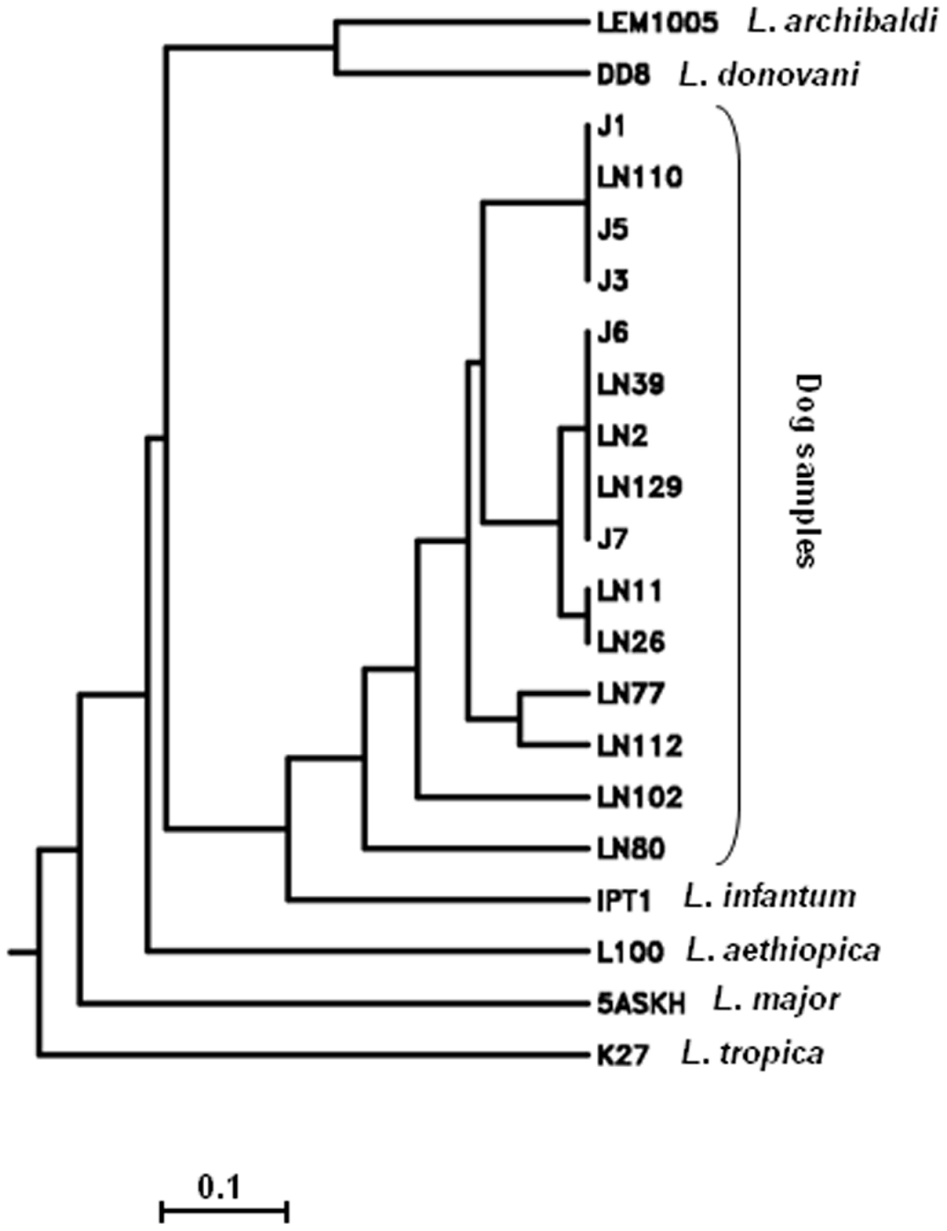
Kitch dendrogram constructed using Nei-modified distances calculated from gp63PCR-RFLP results obtained with lymph node biopsies of dogs from Tunisia. Branches corresponding to Old World representative *Leishmania* strains are indicated.

The gp63PCR products obtained for 15 parasitological positive dogs and representative strains of *L. infantum*, *L. donovani*, *L. archibaldi*, *L. major*, *L. tropica* and *L. aethiopica* species were purified from the gels, digested with *BsiE*I, *Sal*I, *Msc*I, *BsmB*I and *Hinc*II restriction enzymes and analyzed for restriction length polymorphisms by electrophoresis and southern blot analysis using a ^32^P labeled gp63 probe, as previously described [19]. Restriction profiles were polymorphic but species- specific fragments like the presence of an *L. infantum* specific 380 bp *Msc*I fragment and the absence of a 500 bp and a 220 bp *L. donovani* specific *Msc*I fragments [19], allowed identification of the dog *Leishmania* parasites as members of the *L. donovani* complex, more precisely belonging to the *L. infantum* species (Figure 1). In order to better illustrate diversity and phenetic relationships of the amastigotes infecting the studied dogs, the restriction profiles were used to calculate Nei-modified distances and the generated data matrix was then used to construct a dendrogram (Kitch-Margoliash, Phylip package). All dog parasites clustered together with the *L. infantum* reference strain, distinctly from *L. donovani* and *L. archibaldi* representative strains (Figure 2). Whereas, strains representing other Old World species, *L. major*, *L. tropica* and *L. aethiopica* were individualized on separate branches (Figure 2). However, within the dogs' clade, small clusters were observed that were not correlated to epidemiological features like geographical origin, sex or age of dogs. This however highlights occurrence of multiple parasite variants (variability index  = 0.47 (7/15)) characterized by gp63 genes coding for surface antigens having variable, either exposed or buried residues. Three of these variants were shared by 11 parasites (Figure 2). Of relevance to molecular tracking of parasites, different variants were observed in the same transmission area while a same variant was observed in different endemic regions. Moreover, the study here brings information on 2 parasites (LN36 and LN100) that could not be isolated and maintained by *in vitro* culture (Table S1), which illustrates an additional value of the gp63 PCR based assays for molecular diagnosis of canine leishmaniasis.

## Discussion

Variable clinical and biological manifestations characterize *Leishmania* canine infection and leishmaniasis. Its diagnosis still constitutes a major epidemiological problem. Within Tunisian endemic foci, for instance, 50% to 90% of infected dogs are asymptomatic [29], [30], which further show the necessity to use sensitive diagnostic techniques. In spite of their limits, parasitological and serological techniques are still the most common methods used to diagnose canine leishmaniasis and are considered as gold standards [31], [32]. With the advent of molecular tools like PCR, a more sensitive and rapid detection of parasites has become possible. The potential of a gp63 amplification–based tool in molecular diagnosis of canine leishmaniasis was here evaluated. Sensitivity and specificity of this gp63PCR were estimated and compared to parasitological (direct examination and *in vitro* culture), and serological (IFAT) techniques, as well as to other PCR assays. DNAs purified from lymph node (LN) biopsies, taken from 40 Tunisian dogs were PCR amplified in 5 assays targeting, the intra-genic regions of gp63 genes (gp63 PCR), a central region of 18S encoding ribosomal gene (RIB PCR), an *L. infantum* specific repetitive sequence (INF PCR), minicircles of kinetoplast DNA (KIN PCR) and a mammalian phosphorotein gene (PO PCR). This latter PCR was used to verify the absence of inhibitors within the DNA preparations. All PCR products were hybridized to their respective ^32^P labeled probe, which allowed (i) to validate specificity of the obtained signal and (ii) to check for absence of false negative results. An 83.5% sensitivity was calculated for the gp63PCR tool while in controlled laboratory conditions using purified DNA the test could detect 0.01 pg of DNA, which correspond to 0.1 promastigote [19]. This could be explained by differences at the level of amplification efficacy, in relation with parasite burden, presence of host material (including inhibitors) or reaction conditions. Sensitivity of PCR tests was shown to vary outstandingly when applied either on cultured parasites or on peripheral blood samples of infected dogs [18], [33]. Specificity of gp63PCR was estimated on a group of control dogs, constituted by non-infected dogs originating from countries where leishmaniasis is not endemic. Consequently, 100% of specificity was found after EtBr reading. This percentage decreased to a value of 97.8% after hybridization with a ^32^P labeled gp63 probe. Using the same dog sample, 100% of specificity was achieved with EtBr reading for PCRs amplifying genomic sequences (RIB and INF PCRs) while a decrease of specificity (93% for RIB PCR and 89% for INF PCR) was noticed with ^32^P reading. Previous studies that used the INF PCR showed a specificity of 97% when this tool was applied to detect *Leishmania* DNA from patients presenting with visceral leishmaniasis [23]. Here, no certified explanation for the positive (and very faint) signals observed could be provided, travel history of the dogs could be a reason; non–specific amplification or cross-reactivity with other microbes could be other reasons. Nevertheless, it is important to notice that only a few studies have used hybridization with ^32^P labeled probes consecutively after EtBr detection of PCR signals.

Parasitological, serological and molecular techniques, in addition to gp63PCR tool, are methods that showed different rates of infection when tested on our study sample. In other studies that amplified the conserved region of *Leishmania* kDNA minicircles, a prevalence of 67% was found in Spain [16], 24.7% is detected in dogs from an urban area in Brazil [34] while 51.88% was found in western China [35] and 29% in Greece [36]. PCRs using ribosomal genes detected 79.8% of dog infection in southern France [37] and 11.5% in the West Bank, Palestine [38]. However, 58.1% was found in Greece with INF PCR targeting a specific fragment of a repetitive sequence of *L. infantum* DNA [36]. Disparity of the measured rates of infection could be explained by (i) epidemiological differences, (ii) biases introduced when selecting negative dogs or (iii) by efficiency in amplification of different size fragments. Consequently, measure of concordance between results of different tools would allow a better evaluation. Thus, κ concordance values between the different tools showed a substantial and moderate agreement between gp63PCR and parasitological methods (74.6%) and serology IFAT technique (47.4%), respectively, which was higher than that achieved with the other PCR tools. This underscores need for using several methods to diagnose canine infection. Previous studies have in fact shown that a canine infection could be under-estimated when only one technique is used [31], [32]. However, if we consider the appropriate situations where these different techniques could be used, several comments could be advanced. Indeed, although its weak sensitivity, parasitological diagnosis remains a method of choice in individual cases like veterinary consultation, where dogs having patent disease are specifically recruited and for which this kind of examination is sensitive [39]. *In vitro* culture, in these cases, constitutes a limitation, since up to several weeks could be necessary before a result could be advanced. Besides, it is a laborious diagnostic technique mostly performed in research laboratories. It is precisely in these cases that PCR tools are the most useful, since they allow the detection of *Leishmania* DNA with a high sensitivity and specificity, thus providing a rapid diagnosis [32]. Concerning serological methods, these are based on the presence of IgG antibodies within dogs' sera. However, a positive result may indicate an exposure to the parasite but not necessarily an active infection [2], [3]. Moreover, these techniques are not able to reveal the real prevalence, nor the transmission intensity of the infection, since it is difficult to differentiate between an active and a non-active state of the infection [3], [7], [32]. Another challenge in comparative evaluation of tools is the selection of negative control dogs in countries endemic for leishmaniasis. Thus, using simultaneously several diagnostic methods, including PCR, seems to be necessary for canine leishmaniasis diagnosis. In this context, use of gp63PCR (EtBr reading) associated with another PCR tool like RIB PCR, which was most discordant, will allow maximizing possibilities to find positive responses. Gp63 PCR in addition to RIB PCR could constitute a good diagnostic procedure in canine leishmaniasis that would complement parasitological and IFAT methods or constitute alternatives to detect infection. Moreover, a gp63 PCR positive signal was found in the case of 2 dogs, from which *Leishmania* parasites could not be isolated in culture. This further emphasized the interesting potential of this gp63PCR as a molecular tool, able of detecting and studying parasites, bypassing *in vitro* culture steps. On the other hand, the gp63 assay did not detect parasites of cases that were positive by the classical techniques. Presence of specific inhibitors to this assay or parasites having polymorphic priming sites may be causes affecting PCR efficiency. PO primers amplified all the Tunisian dog samples inferring that other causes than PCR inhibition may explain this sensitivity default.

Intra-specific polymorphism of parasites within infected dogs' lymph nodes was evaluated by RFLP analysis of the amplified gp63PCR fragments by several restriction enzymes [19] followed by phenetic analysis using Nei-modified distances. The dendrogram confirmed the identity of the studied dog parasites as belonging to the *L. donovani* complex and more precisely to the *L. infantum* species. Polymorphic PCR-RFLP profiles highlighted their genetic variability constituting in some cases small groups that clustered together on the dendrogram. However this variability did not correlate with epidemiological features like geographical origin, sex or age of dogs. Gp63 PCR-RFLP highlighted geographical structuring of *L. infantum* and *L. donovani* parasites and importance of host selection pressures were hypothesized [19]. Thus, it appears important to develop further studies comparing parasites having diverse host origins. Nevertheless, this gp63PCR constitutes an innovative approach that allows study of *L. infantum* variability within the reservoir to assess occurrence of parasite variants in a concomitant way to their detection.

## Supporting Information

Table S1 **Panel of dogs collected from leishmaniasis endemic regions in Tunisia and results of parasitology, serology and PCR investigations.**
(DOCX)

Table S2 **Panel of control dogs collected from non-endemic countries for leishmaniasis and PCR results.**
(DOCX)
