# Supplementary material for: Evaluation of a gp63–PCR Based Assay as a Molecular Diagnosis Tool in Canine Leishmaniasis in Tunisia
Source: PLoS One. 2014 Aug 25;9(8):e105419. doi: 10.1371/journal.pone.0105419 (PMC4143256; doi:10.1371/journal.pone.0105419)
Supplement: Table S1 — Panel of dogs collected from leishmaniasis endemic regions in Tunisia and results of parasitology, serology and PCR investigations. (DOCX) [file pone.0105419.s001.docx]

Table S1. Panel of dogs collected from leishmaniasis endemic regions in Tunisia and their parasitology, serology and PCR investigations results.

|  |  |  |  |  |  |  |  |  |  |  |  |  |  |  |  |
| --- | --- | --- | --- | --- | --- | --- | --- | --- | --- | --- | --- | --- | --- | --- | --- |
| Number^a^ | **Tunisian dogs** | **Geographic origin** | **Sex** | **Age** | **Parasitology** | **Serology (IFAT)** | **PO PCR^b^** | **RIB PCR^c^** | | **INF PCR^c^** | | **KIN PCR^c^** | | **gp63 PCR^c^** | |
|  |  |  |  |  |  |  |  | **EtBr** | **^32^P** | **EtBr** | **^32^P** | **EtBr** | **^32^P** | **EtBr** | **^32^P** |
| 1 | LN112 | Kasserine | M | 1.00 | + | + | + | + | + | + | + | + | + | + | + |
| 2 | LN129 | Goubellat | M | 2.00 | + | + | + | - | + | + | + | + | + | + | + |
| 3 | LN26 | Goubellat | M | 2.50 | + | + | + | + | + | + | + | + | + | + | + |
| 4 | LN11 | Kasserine | M | 4.00 | + | + | + | - | - | + | + | - | + | + | + |
| 5 | LN80 | Goubellat | F | 4.00 | + | - | + | + | + | + | + | + | + | + | + |
| 6 | LN2 | Kasserine | F | 10.00 | + | + | + | - | + | + | + | - | - | + | + |
| 7 | LN39 | Kasserine | F | 8.00 | + | + | + | + | + | + | + | - | + | + | + |
| 8 | LN69 | Goubellat | M | 8.00 | + | + | + | + | + | + | + | + | + | - | - |
| 9 | LN77 | Goubellat | M | 8.00 | + | + | + | + | + | + | + | + | + | + | + |
| 10 | LN102 | Kasserine | M | 8.00 | + | + | + | + | + | + | + | - | - | + | + |
| 11 | LN110 | Kasserine | M | 8.00 | + | + | + | + | + | + | + | - | + | + | + |
| 12 | LN J1 | Salambo | M | 4.00 | + | + | + | - | - | - | + | + | + | + | + |
| 13 | LN J2 | Tunis | M | 6.00 | + | + | + | - | - | - | + | - | - | - | - |
| 14 | LN J3 | Le Kram | M | 7.50 | + | + | + | - | + | + | + | + | + | + | + |
| 15 | LN J4 | Soliman | M | 6.00 | + | + | + | - | + | - | + | - | + | - | - |
| 16 | LN J5 | Ariana | M | 2.00 | + | + | + | - | + | + | + | + | + | + | + |
| 17 | LN J6 | Ariana | F | 4.00 | + | + | + | + | + | + | + | + | + | + | + |
| 18 | LN J7 | Ariana | M | 2.00 | + | + | + | + | + | + | + | + | + | + | + |
| 19 | LN121 | Goubellat | F | 0.30 | - | - | + | - | + | - | + | - | + | - | - |
| 20 | LN124 | Goubellat | F | 0.25 | - | - | + | + | + | - | + | - | + | - | - |
| 21 | LN32 | Kasserine | F | 2.00 | - | - | + | - | + | - | + | - | + | - | - |
| 22 | LN84 | Goubellat | M | 2.00 | - | + | + | + | + | - | + | + | + | - | - |
| 23 | LN96 | Kasserine | F | 2.50 | - | - | + | + | + | + | + | - | + | - | - |
| 24 | LN140 | Goubellat | M | 3.00 | - | - | + | + | + | - | + | + | + | - | - |
| 25 | LN86 | Goubellat | F | 6.00 | - | + | + | + | + | + | + | + | + | - | - |
| 26 | LN142 | Goubellat | M | 6.00 | - | - | + | - | + | - | + | - | + | - | - |
| 27 | LN10 | Kasserine | M | 10.00 | - | - | + | - | + | - | + | - | + | - | - |
| 28 | LN36 | Kasserine | F | 8.00 | - | + | + | + | + | + | + | + | + | + | + |
| 29 | LN49 | Kasserine | M | 8.00 | - | - | + | + | + | - | + | - | + | - | - |
| 30 | LN74 | Goubellat | M | 8.00 | - | - | + | + | + | - | + | - | + | - | - |
| 31 | LN100 | Kasserine | M | 8.00 | - | + | + | + | + | + | + | + | + | + | + |
| 32 | LN105 | Kasserine | M | 8.00 | - | - | + | + | + | + | + | + | + | - | - |
| 33 | LN107 | Kasserine | F | 8.00 | - | - | + | + | + | + | + | + | + | - | - |
| 34 | LN122 | Goubellat | F | 8.00 | - | - | + | + | + | + | + | + | + | - | - |
| 35 | LN139 | Goubellat | M | 8.00 | - | - | + | + | + | + | + | + | + | - | - |
| 36 | LN66 | Goubellat | F | 0.50 | - | + | + | + | + | - | + | - | - | - | - |
| 37 | LN3 | Kasserine | M | 4.00 | - | + | + | - | - | - | + | - | + | - | - |
| 38 | LN99 | Kasserine | M | 8.00 | - | + | + | + | + | - | - | - | - | - | - |
| 39 | LN143 | Goubellat | F | 8.00 | - | + | + | + | + | + | + | - | - | - | - |
| 40 | LN28 | Goubellat | M | 12.00 | - | + | + | - | - | - | + | - | - | - | - |
| Sensitivity^d^ (%) | |  | |  |  |  |  | 55.6 (10/18) | 83.5 (15/18) | 83.5 (15/18) | 100 (18/18) | 61.1 (11/18) | 83.5 (15/18) | 83.5 (15/18) | 83.5 (15/18) |
| Infection rate^e^ (%) | |  | |  | 45.0 (18/40) | 65.0 (26/40) |  | 87.5 (35/40) | | 97.5 (39/40) | | 82.5 (33/40) | | 42.5 (17/40) | |

^a^ dogs from number 1 to 18 constitute the positive control group which are parasitologically confirmed dogs (smear examination or culture).

^b^ PO PCR targets a mammalian mitochondrial phosphoprotein gene.

^c^ RIB, INF, KIN and gp63 PCRs target a central region of 18S ribosomal gene, a repetitive genomic sequence, minicircles of the kinetoplastic DNA and gp63 family coding sequences, respectively in *Leishmania*.

^d^ sensitivity of the different PCR assays corresponds to the proportion of positive dogs among the 18 parasitologically confirmed ones.

^e^ infection rates calculated for each tool used are the proportion of positive dogs among the total dog number.

Abbreviations: LN, lymph node; F, female; M, male; EtBr, Ethidium bromide staining and reading under UV light; ^32^P, autoradiographic reading after hybridization with a ^32^P labeled probe; + and –, presence and absence of an amplified product, respectively.
