# Supplementary material for: Evaluation of a gp63–PCR Based Assay as a Molecular Diagnosis Tool in Canine Leishmaniasis in Tunisia
Source: PLoS One. 2014 Aug 25;9(8):e105419. doi: 10.1371/journal.pone.0105419 (PMC4143256; doi:10.1371/journal.pone.0105419)
Supplement: Table S2 — Panel of control dogs collected from non-endemic countries for leishmaniasis and PCR results. (DOCX) [file pone.0105419.s002.docx]

Table S2. Panel of control dogs collected from non-endemic countries for leishmaniasis and their PCR results.

|  |  |  |  |  | |  | |  | |  | |
| --- | --- | --- | --- | --- | --- | --- | --- | --- | --- | --- | --- |
| Control dogs (N=45) | **Breed** | **Country of origin** | **PO PCR^a^** | **RIB PCR^b^** | | **INF PCR^b^** | | **KIN PCR^b^** | | **gp63 PCR^b^** | |
|  |  |  |  | **EtBr** | **^32^P** | **EtBr** | **^32^P** | **EtBr** | **^32^P** | **EtBr** | **^32^P** |
| T99 | Welsh corgi | Great Britain | + | **–** | **–** | **–** | **–** | **–** | **–** | **–** | **–** |
| T100 | Welsh corgi | The Netherlands | + | **–** | **–** | **–** | **–** | **–** | **–** | **–** | **–** |
| T101 | Welsh corgi | The Netherlands | + | **–** | **–** | **–** | **–** | **–** | **–** | **–** | **–** |
| T102 | Welsh corgi | The Netherlands | + | **–** | **–** | **–** | **–** | **–** | **–** | **–** | **–** |
| T103 | Welsh corgi | The Netherlands | + | **–** | **–** | **–** | **–** | **–** | **–** | **–** | **–** |
| T104 | Welsh corgi | The Netherlands | + | **–** | **–** | **–** | **+** | **–** | **–** | **–** | **–** |
| T106 | Welsh corgi | Finland | + | **–** | **–** | **–** | **–** | **–** | **–** | **–** | **–** |
| T107 | Welsh corgi | Finland | + | **–** | **–** | **–** | **–** | **–** | **–** | **–** | **–** |
| T108 | Welsh corgi | Finland | + | **–** | **+** | **–** | **–** | **–** | **–** | **–** | **–** |
| T109 | Welsh corgi | Germany | + | **–** | **–** | **–** | **–** | **–** | **–** | **–** | **–** |
| T115 | Welsh corgi | Australia | + | **–** | **–** | **–** | **–** | **–** | **–** | **–** | **–** |
| T116 | Welsh corgi | Australia | + | **–** | **–** | **–** | **–** | **–** | **–** | **–** | **–** |
| T117 | Welsh corgi | Australia | + | **–** | **–** | **–** | **–** | **–** | **–** | **–** | **–** |
| T118 | Welsh corgi | Australia | + | **–** | **–** | **–** | **–** | **–** | **–** | **–** | **–** |
| T119 | Welsh corgi | Australia | + | **–** | **–** | **–** | **–** | **–** | **–** | **–** | **–** |
| W18 | Wolfhound | Great Britain | + | **–** | **–** | **–** | **–** | **–** | **–** | **–** | **–** |
| W19 | Wolfhound | Great Britain | + | **–** | **–** | **–** | **–** | **–** | **–** | **–** | **–** |
| W20 | Wolfhound | Great Britain | + | **–** | **–** | **–** | **–** | **–** | **–** | **–** | **–** |
| W21 | Wolfhound | Great Britain | + | **–** | **–** | **–** | **–** | **–** | **–** | **–** | **–** |
| W22 | Wolfhound | Sweden | + | **–** | **–** | **–** | **–** | **–** | **–** | **–** | **–** |
| W23 | Wolfhound | Sweden | + | **–** | **–** | **–** | **–** | **–** | **–** | **–** | **–** |
| W25 | Wolfhound | Great Britain | + | **–** | **–** | **–** | **–** | **–** | **–** | **–** | **–** |
| W26 | Wolfhound | Great Britain | + | **–** | **–** | **–** | **–** | **–** | **–** | **–** | **–** |
| W27 | Wolfhound | Great Britain | **–** | **–** | **–** | **–** | **+** | **–** | **–** | **–** | **–** |
| W28 | Wolfhound | Great Britain | + | **–** | **–** | **–** | **–** | **–** | **–** | **–** | **–** |
| IS11 | Irish setter | Great Britain | + | **–** | **–** | **–** | **–** | **–** | **–** | **–** | **–** |
| IS12 | Irish setter | Great Britain | + | **–** | **+** | **–** | **–** | **–** | **–** | **–** | **–** |
| IS13 | Irish setter | Great Britain | + | **–** | **–** | **–** | **–** | **–** | **–** | **–** | **–** |
| IS14 | Irish setter | Great Britain | + | **–** | **–** | **–** | **–** | **–** | **–** | **–** | **–** |
| IS15 | Irish setter | Great Britain | **–** | **–** | **–** | **–** | **+** | **–** | **–** | **–** | **–** |
| IS16 | Irish setter | Great Britain | + | **–** | **–** | **–** | **–** | **–** | **–** | **–** | **–** |
| IS17 | Irish setter | Great Britain | + | **–** | **–** | **–** | **–** | **–** | **–** | **–** | **–** |
| IS18 | Irish setter | Great Britain | + | **–** | **–** | **–** | **–** | **–** | **–** | **–** | **–** |
| IS19 | Irish setter | Great Britain | + | **–** | **–** | **–** | **–** | **–** | **–** | **–** | **–** |
| IS20 | Irish setter | Great Britain | + | **–** | **–** | **–** | **–** | **–** | **–** | **–** | **–** |
| C16 | Cocker spaniel | Great Britain | + | **–** | **–** | **–** | **–** | **–** | **–** | **–** | **–** |
| C17 | Cocker spaniel | Great Britain | + | **–** | **–** | **–** | **–** | **–** | **–** | **–** | **–** |
| C18 | Cocker spaniel | Great Britain | **–** | **–** | **–** | **–** | **–** | **–** | **–** | **–** | **–** |
| C19 | Cocker spaniel | Great Britain | + | **–** | **–** | **–** | **+** | **–** | **–** | **–** | **–** |
| C20 | Cocker spaniel | Great Britain | + | **–** | **–** | **–** | **–** | **–** | **–** | **–** | **–** |
| L29 | Labrador | Great Britain | + | **–** | **–** | **–** | **–** | **–** | **–** | **–** | **–** |
| L30 | Labrador | Great Britain | + | **–** | **–** | **–** | **–** | **–** | **–** | **–** | **+** |
| L31 | Labrador | Great Britain | + | **–** | **+** | **–** | **–** | **–** | **–** | **–** | **–** |
| L32 | Labrador | Great Britain | + | **–** | **–** | **–** | **+** | **–** | **–** | **–** | **–** |
| L33 | Labrador | Great Britain | + | **–** | **–** | **–** | **–** | **–** | **–** | **–** | **–** |
| Specificity^c^ (%) | |  |  | 100 | 93.3 (42/45) | 100 | 88.9 (40/45) | 100 | 100 | 100 | 97.8 (44/45) |

^a^ PO PCR targets a mammalian mitochondrial phosphoprotein gene.

^b^ RIB, INF, KIN and gp63 PCRs target a central region of 18S ribosomal gene, a repetitive genomic sequence, minicercles of the kinetoplastic DNA and gp63 family coding sequences, respectively in *Leishmania*.

^c^ specificity of the different PCR assays corresponds to the number of negative dogs among the control dog group.

Abbreviations: EtBr, Ethidium bromide staining and reading under UV light; ^32^P, autoradiographic reading after hybridization with a ^32^P labeled probe; + and –, presence and absence of an amplified product, respectively.
